# A Review of Allogeneic Hematopoietic Stem Cell Transplantation in Metastatic Breast Cancer

**Published:** 2018-04-01

**Authors:** Nuri Karadurmus, Ugur Sahin, Bilgin Bahadir Basgoz, Fikret Arpaci, Taner Demirer

**Affiliations:** 1 Gulhane Military Medical Academy, Department of Medical Oncology, Etlik, Ankara, Turkey; 2 Ankara University Medical School, Department of Hematology, Ankara, Turkey; 3 Liv Hospital, Department of Medical Oncology, Ankara, Turkey

**Keywords:** Metastatic breast cancer, Allogeneic hematopoietic stem cell transplantation

## Abstract

Breast cancer (BC) has a high mortality rate and metastatic BC is almost incurable despite hormonal therapy and chemotherapy. The second and third lines of chemotherapies usually yield transient responses and the median survival is generally as low as 18-24 months. Autologous and allogeneic hematopoietic stem cell transplantation (HSCT) have been extensively investigated in this setting. The presence of immune mediated anti-tumor effects referred to as graft-versus-tumor (GvT) effects after allogeneic HSCT among patients with solid tumors have been clearly defined. The advantages of allogeneic HSCT over autologous HSCT for metastatic BC are i) cancer-free graft and ii) immune-mediated GvT effects mediated by human leukocyte antigen compatible donor T-cells. In conclusion, a GvT effect does exist against metastatic BC and play a key role in tumor response. This review aims to describe the background, rationale, and clinical results of allogeneic HSCT as a potential alternative treatment in metastatic BC.

## Introduction

 Breast cancer (BC) is the most common cancer among women and the second most common cause of cancer death in women, accounting for 40.000 deaths annually in the United States^   1 ^. Metastatic BC is almost incurable despite hormonal therapy and chemotherapy^2^^,^^3^. The second and third lines of chemotherapies usually yield transient responses and the median survival is generally as low as 18-24 months^        4 ^. Due to this limited efficacy of chemotherapy, the development of novel therapeutic strategies to currently available modalities has become essential.

Higher dose – better response concept has been accepted as a treatment strategy in 1980s and high-dose chemotherapy (HDC) has been used for metastatic BC since then^5^^-^^9^. However, increasing the chemotherapy dose in advanced BC has not been reported to be beneficial, neither on overall survival (OS)^10^^-^^12^ nor relapse-free survival (RFS)^   13 ^.

Regimens employing autologous hematopoietic stem cell transplantation (HSCT) following HDC have been developed in order to avoid myelotoxic effects of HDC^14^^-^^20^. Although this approach is expected to increase survival by optimizing tumor response^21^^-^^25^, its efficacy is controversial. A meta-analysis of 15 well-known randomized trials of HDC plus autologous HSCT for high-risk primary BC reported a benefit in RFS, but not OS^        26 ^. Patient’s age^   27 ^, hormone receptor status^28^^-^^30^, tumor grade^30^^, ^^31^ and lymph node involvement^        32 ^ had statistically significant interactions with OS^        26 ^. RFS was significantly better among younger patients with HDC. The possible reasons of disease recurrence after HSCT might be the contamination of malignant cells during autologous stem cell harvest as well as incomplete eradication of disease^        33 ^.

Allogeneic HSCT is primarily used in patients with relapsed or high-risk hematologic malignancies^21^^,^^34^ and the efficacy of this treatment has been substantially demonstrated^   35 ^. The principles of allogeneic HSCT consist of maximal tumor cytoreduction with high-dose chemoradiotherapy and adequate immunosuppression in order to provide engraftment of donor stem cells as well as graft-versus-tumor (GvT) effect^   36 ^. The controversial and disappointing results of studies investigating high-dose chemotherapy with autologous stem cell rescue in patients with solid tumors^9^^, ^^18^^-^^20^^, ^^37^^-^^40^ have led to development of novel approaches such as adoptive T-cell therapies (ATCT), targeted therapies and allogeneic HSCT with reduced-intensity conditioning (RIC) regimens, which aim to create and take advantage of a GvT effect in order to induce more durable responses^36^^, ^^41^.

Non-myeloablative (NMA) and RIC regimens for allogeneic HSCT have introduced a new era for treating elderly and those with comorbidities. These regimens are currently being used for as much as 40% of all allogeneic HSCTs and are becoming increasingly popular. The growing knowledge on the immune system and T-cell biology has made allogeneic HSCT a promising approach for the treatment of some solid tumors^36^^,^^42^^-^^44^. Several phase I and II studies, which were conducted by the European Society for Blood and Marrow Transplantation Solid Tumors Working Party (EMBT-STWP) documented the presence of a GvT effect in patients with various solid tumorssuch as renal, ovarian, breast and colon cancers and soft tissue sarcomas^36^^, ^^42^^-^^44^.

The successful engraftment rates together with a lower transplant-related mortality and the presence of GvT effect made allogeneic HSCT with RIC an attractive option for the treatment of several solid tumors within the last decade^36^^,^^42^^-^^47^. The lower toxicity obtained by the reduction of chemo-radiotherapy dose also enables allogeneic HSCT with RIC to become a choice for the elderly and medically fragile patients with metastatic solid tumors^36^^, ^^42^^-^^47^.

The first report of allogeneic HSCT in metastatic BC had been published by Eibl et al. in 1996 and successfully demonstrated the immune mediated anti-tumor effects referred to as GvT effects among patients with solid tumors^        41 ^. Subsequently, several series that focused on GvT effect after allogeneic HSCT in many advanced solid tumors^36^^, ^^42^^-^^44^^, ^^48^as well as metastatic BC had been published^49^^-^^52^.

This review aims to describe the background, rationale and clinical results of allogeneic HSCT as an alternative treatment in metastatic BC.

**Graft-versus-tumor effect in breast cancer**

Graft versus leukemia effect was first demonstrated in a leukemiac murine model that received allogeneic HSCT from other strains of mice following high-dose body irradiation^   53 ^. Later, GvT effect in a solid tumor after allogeneic BMT has also been demonstrated in a murine model in 1984^   54 ^.Morecki*et al*. demonstrated a GvT effect in mice implanted with 4T1 mammary carcinoma cell line and given minor histocompatibility mismatched DBA/2 spleen cells^   55 ^. This direct GvT effect mediated by the alloreactive donor splenocytes in the absence of any anti-carcinoma agents has also been demonstrated by direct inhibition of liver metastases through intraportal inoculation of allogeneic splenocytes, but not syngeneic splenocytes^   56 ^.

The advantages of allogeneic HSCT over autologous HSCT for metastatic BC are i) cancer-free graft and ii) immune-mediated GvT effects mediated by human leukocyte antigen(HLA)-compatible donor T-cells^33^^,^^36^. These immune-mediated effects led to a transition from a chemotherapy-based approach to an immunotherapy-based approach in the management of BC^        57 ^.The switch from targeting maximal tumor cytoreduction via HDC to induction of immune GVT effects also gave rise to development of RIC and NMA regimens instead of conventional myeloablative conditioning regimens ^41^^,^^49^. RIC regimens substantially reduced the high transplant-related morbidity and mortality^37^^-^^40^^,^^58^ while allowing for a complete myeloid/lymphoid engraftment.^[^^45^^-^^47^^, ^^50^^-^^52^^]^ As a result, allogeneic HSCT may become an appropriate treatment option for the elderly and medically fragile patients with metastatic BC^41^^, ^^49^.

## Results

 After demonstration of tumor regression in metastatic BC via allogeneic T-lymphocyte mediated GvT effects in several murine models^59^^,^^60^, the National Cancer Institute of the National Institutes of Health in the USA investigated whether a clinical graft-versus-BC effect existed via allogeneic lymphocytes after allogeneic HSCT from HLA-matched siblings following a RIC regimen^   61 ^. The study included 16 metastatic BC patients who had progressed after chemotherapy and hormonal therapy. In order to avoid the overlap of immunological GvT effect and anti-tumor effect of cytotoxic chemotherapy used in the pre-transplant conditioning regimen, allogeneic T-lymphocytes were removed from the stem cell graft and were subsequently administered at escalating doses after allogeneic HSCT (on +42, +70, and +98 days). Objective tumor regression occurred in six patients 28 days after allogeneic HSCT. Disease progression following allogeneic HSCT was observed before subsequent tumor regression in 2 of the 6 patients. Tumor regressions obtained simultaneously with the accomplishment of complete donor T-lymphoid engraftment associated with the development of graft-versus-host disease (GvHD) and abrogated after systemic immunosuppression^        52 ^.

Another study evaluated HDC plus autologous HSCT combined with allogeneic HSCT following RIC regimen^        62 ^. With this strategy, Carella, et al. aimed to achieve maximum tumor reduction prior to allogeneic HSCT. This approach may provide the benefits of a conventional allograft while reducing the typical acute toxicities and myeloablative therapy associated mortality. The study enrolled 17 metastatic BC patients with a median age of 41 years. The primary endpoint of the study was to decrease the non-relapse mortality (NRM), which is currently reported as 20-35% following allografting with NMA. Donor engraftment was established in 13 patients. Partial remission after HDC plus autologous HSCT was present in 3 patients who achieved complete remission after the development of GvHD following allogeneic HSCT with RIC. Grade II or higher acute GvHD was observed in 5 patients (29%) and chronic GvHD requiring systemic immunosuppression in 6 patients (5 had extensive involvement). No NRM-related death occurred in the first 100 days after allogeneic HSCT. At a median of 1320 days (range 90-2160) after allogeneic HSCT, 5 patients (29%) were alive (3 achieved complete remission and 2 had stable disease).

Ueno et al. analyzed the outcomes of 66 poor-risk metastatic BC patients who had undergone allogeneic HSCT in 15 centers of the Center for International Blood and Marrow Transplant Research (CIBMTR) or EBMT and compared the outcomes and toxicities of conventional myeloablative conditioning (MAC) regimens to RIC regimens^        33 ^. MAC and RIC regimen was given to 39 (59%) and 27 (41%) patients, respectively. Despite including a significantly higher number of patients with poor prognostic features before the transplant (63% vs. 26%, p<0.002), treatment-related mortality was lower in the RIC group (7% vs 29% at 100 days, p< 0.03). RFS at 1 year was higher in the MAC group, but it did not reach statistical significance (23% vs 8%, p<0.09). Consistent with a GvT effect, patients who developed acute GvHD following RIC regimen had lower relapse or progression risk than those who did not (p<0.03); however, this did not translate into a RFS advantage^[^^33^^]^. Immune manipulation such as donor lymphocyte infusion (DLI) for persistent or progressive disease was performed in 9 out of 33 patients (27%) and led to disease response or stable disease. Authors concluded that preclinical and clinical studies are needed in order to facilitate targeted adoptive immunotherapy to explore the benefit of a GvT effect in breast cancer.^[^^33^^]^ 

Despite its great potential, ATCT for cancer control still has a marginal role in the management of patients with solid tumors although its use in the setting of melanoma seems ready for development as a routine therapy^   63 ^. Indeed, the extensive infra-structure needed for exploiting ATCT still restricts its use to academic centers with specific programs in the field. We have to emphasize that the major obstacle for a wider application of ATCT to treat human cancer is the personalized nature of the approach^   63 ^. More clinical studies will result in deployment of innovative allogeneic HSCT approaches that take advantage of GvT effects to control disease while minimizing the treatment- related mortality or scale of GvHD. Future studies should include patients with better performance status and with chemotherapy responsive disease before transplant in order to obtain a maximum benefit from GvT effects.

In conclusion, a GvT effect does exist against metastatic BC and may play a key role in tumor response. If conditioning regimen-related toxicities are reduced and response rates are increased via advances in innovative treatments such as immunotherapy, adoptive T-cell therapies (ATCT) and targeted therapies, this treatment modality might be included in the armamentarium of treatments for BC^   63 ^.
